# Shorter telomere length as a prognostic marker for survival and recurrence in breast cancer: a systematic review and meta-analysis

**DOI:** 10.37349/etat.2025.1002289

**Published:** 2025-02-13

**Authors:** Dhyas Munandar Arya Sasmita, Kavi Gilang Permana, Teguh Aryandono, Didik Setyo Heriyanto, Sumadi Lukman Anwar

**Affiliations:** IRCCS Istituto Romagnolo per lo Studio dei Tumori (IRST) “Dino Amadori”, Italy; ^1^Faculty of Medicine, Public Health, and Nursing, Universitas Gadjah Mada, Yogyakarta 55281, Indonesia; ^2^Department Surgery, Dr. Soeradji Tirtonegoro General Hospital, Klaten 57234, Indonesia; ^3^Department Oncological Surgery, Dr. Sardjito General Hospital, Yogyakarta 55281 Indonesia; ^4^Department of Anatomical Pathology, Dr. Sardjito General Hospital, Yogyakarta 55281, Indonesia

**Keywords:** Telomere length, breast cancer, prognosis, survival, recurrence

## Abstract

**Background:**

Telomere length is a potential prognostic biomarker in breast cancer, but its clinical utility remains uncertain due to inconsistent findings across the literature. This systematic review and meta-analysis aims to evaluate the association between telomere length and breast cancer survival outcomes, including overall survival (OS), disease-specific survival (DSS), disease-free survival (DFS), and recurrence-free survival (RFS).

**Methods:**

A systematic search of ten sources, including databases and publishers (JSTOR, Nature, ProQuest, PubMed, Sage Journals, ScienceDirect, Science, Scopus, Springer, and Wiley) was conducted to identify studies published up to December 31, 2023. Studies reporting associations between telomere length and survival outcomes in breast cancer patients were included. Hazard ratios (HRs) and odds ratios (ORs) with 95% confidence intervals (CI) were extracted or calculated. Quality assessment was performed using the Newcastle-Ottawa Scale, and publication bias was evaluated using funnel plots, Egger’s, and Begg’s tests.

**Results:**

Nine studies involving 3,145 breast cancer patients were included. Shorter telomere length was significantly associated with increased recurrence risk (DFS/RFS) (pooled HR: 1.97; 95% CI: 1.04–3.74, *P* = 0.039), indicating a nearly twofold increase in risk. Trends toward worse OS (pooled HR: 1.60; 95% CI: 0.90–2.86, *P* = 0.110) and DSS (pooled HR: 1.09; 95% CI: 0.80–1.49, *P* = 0.565) were observed, but did not reach statistical significance. Additionally, shorter telomere length was significantly associated with premenopausal status (pooled OR: 1.34; 95% CI: 1.06–1.70, *P* = 0.01).

**Discussion:**

Shorter telomere length is associated with an increased risk of recurrence in breast cancer, highlighting its potential as a prognostic biomarker. However, further research is needed to standardize telomere length measurement methodologies and validate these findings across diverse populations and breast cancer subtypes.

## Introduction

Breast cancer remains the most prevalent malignancy and a leading cause of cancer-related mortality among women globally, with over 2.3 million new cases and nearly 685,000 deaths annually [1]. Despite significant advances in early detection and treatment modalities, prognostic outcomes continue to vary widely due to the inherent heterogeneity of the disease and differences in tumor biology [2]. While established prognostic factors such as tumor stage, molecular subtype, and hormone receptor status provide valuable insights, there is a growing need for additional biomarkers to enhance risk stratification and guide personalized treatment decisions [3, 4].

Telomere length has emerged as a potential biomarker of cancer prognosis due to its fundamental role in maintaining genomic stability [5]. Telomeres, repetitive nucleotide sequences (TTAGGG) located at the ends of chromosomes, protect genomic integrity by preventing chromosomal end-to-end fusions and instability [6]. Telomeres progressively shorten with each cell division due to incomplete DNA replication, a process further accelerated by oxidative stress and chronic inflammation [7, 8]. This progressive shortening can lead to genomic instability, cellular senescence, and oncogenesis [9]. In cancer, telomere dysfunction is associated with tumor progression, metastasis, and poor clinical outcomes [10, 11].

Short telomeres are associated with increased genomic instability, a hallmark of cancer progression, and poor outcomes in patients with breast cancer [12, 13]. Research on specific subtypes reveals telomere shortening in aggressive forms of breast cancer, including triple-negative breast cancer (TNBC) and human epidermal growth factor receptor 2 (HER2)-positive breast cancer [14–16]. Although numerous studies have explored the relationship between telomere length and breast cancer outcomes, findings remain inconsistent. Some studies report shorter telomeres as a risk factor for worse survival [17–22], while others demonstrate weak or non-significant associations [23–25]. These discrepancies are likely due to variations in telomere measurement techniques, differences in sample types, and population heterogeneity [26]. Furthermore, population-specific differences in telomere biology and genetic predispositions, such as *BRCA2* mutations, have been shown to increase the risk of breast cancer development and progression, adding another layer of complexity to prognostic interpretations [27].

This systematic review and meta-analysis aims to evaluate the association between telomere length and breast cancer survival outcomes, including overall survival (OS), disease-specific survival (DSS), disease-free survival (DFS), and recurrence-free survival (RFS). By integrating data from multiple studies, this research seeks to clarify the prognostic significance of telomere length and identify gaps that must be addressed to advance its clinical utility.

## Materials and methods

### Study design and protocol

This systematic review and meta-analysis adhered to the Preferred Reporting Items for Systematic Reviews and Meta-Analysis Protocols (PRISMA-P) guidelines [28]. The study protocol was registered in the International Prospective Register of Systematic Reviews (PROSPERO) (Registration ID: CRD42023436764). Ethical approval and informed consent were not required as this study involved the secondary analysis of published data without direct patient interaction.

### Eligibility criteria

Studies were included if they met the following criteria: (1) patients with histologically or pathologically confirmed breast cancer; (2) patients categorized based on telomere length (short vs. long); (3) studies reporting survival outcomes, including OS, DSS, DFS, or RFS, presented as hazard ratios (HRs) with 95% confidence intervals (CI), Kaplan-Meier curves, or raw data suitable for extraction; (4) observational studies (cohort or case-control) published in English.

Studies were excluded if they were reviews, conference abstracts, editorials, in vitro or in vivo studies, or lacked sufficient data. Sufficient data refers to clearly reported HR and CI or data extractable from Kaplan-Meier curves [29].

### Search strategy

A comprehensive literature search was conducted across databases and publishers, including JSTOR, Nature, ProQuest, PubMed, Sage Journals, ScienceDirect, Science, Scopus, Springer, and Wiley, for studies published up to December 31, 2023. Search terms combined variations of keywords such as “Breast Cancer”, “Breast Neoplasm”, “Breast Tumor”, “Breast Malignant Neoplasms”, “Mammary Carcinoma”, “Breast Carcinoma”, “Telomere Length”, “Prognosis”, “Survival”, and “Recurrence”. Specific search strategies for each database are provided in Table S1.

### Study selection

Two independent authors screened the titles and abstracts of all retrieved articles. Full-text assessments were performed to confirm eligibility based on predefined criteria. Discrepancies were resolved through consensus or consultation with another author. The study selection process is illustrated in the PRISMA flowchart.

### Data extraction

Data were extracted independently by two authors using a standardized form. Extracted information included: (1) Study characteristics: first author, publication year, geographic region, sample type, telomere measurement metric, method, telomere length categorization, cut-off value, and follow-up duration. (2) Patient characteristics: menopausal status, tumor grade, tumor stage, lymph node involvement, and hormone receptor status; (3) Outcomes: survival metrics (OS, DSS, DFS, RFS), HR, odds ratio (OR), 95% CI, and *P*-values. For studies without directly reported HR or 95% CI, data were extracted from Kaplan-Meier curves using validated methods [29].

### Quality assessment

Study quality was assessed using the Newcastle-Ottawa Scale (NOS), which evaluates selection, comparability, and outcome assessment [30]. Studies scoring ≥ 7 were considered high-quality. Disagreements in scoring were resolved through discussion or input from other authors.

### Statistical analyses

Statistical analyses were conducted using STATA v.17. The following approaches were applied: (1) effect size calculation: pooled HR with 95% CI for survival outcomes (OS, DSS, DFS, RFS) and pooled OR for clinicopathological characteristics; (2) heterogeneity: assessed using Cochran’s Q test and Higgins *I*^2^ statistic. A random-effects model was applied if heterogeneity was significant (*I*² > 50%), otherwise a fixed-effects model was used [31]; (3) subgroup analyses: performed based on geographic region, telomere measurement metric, method, and sample type. Only subgroups with at least three studies were included in the analysis; (4) publication bias: evaluated using funnel plots [32], Egger’s test [33], and Begg’s test [34]. Funnel plot asymmetry was interpreted cautiously given the small number of studies included; (5) sensitivity analysis: conducted by systematically excluding individual studies to assess the robustness of pooled estimates.

## Results

### Study selection

**Figure 1:**
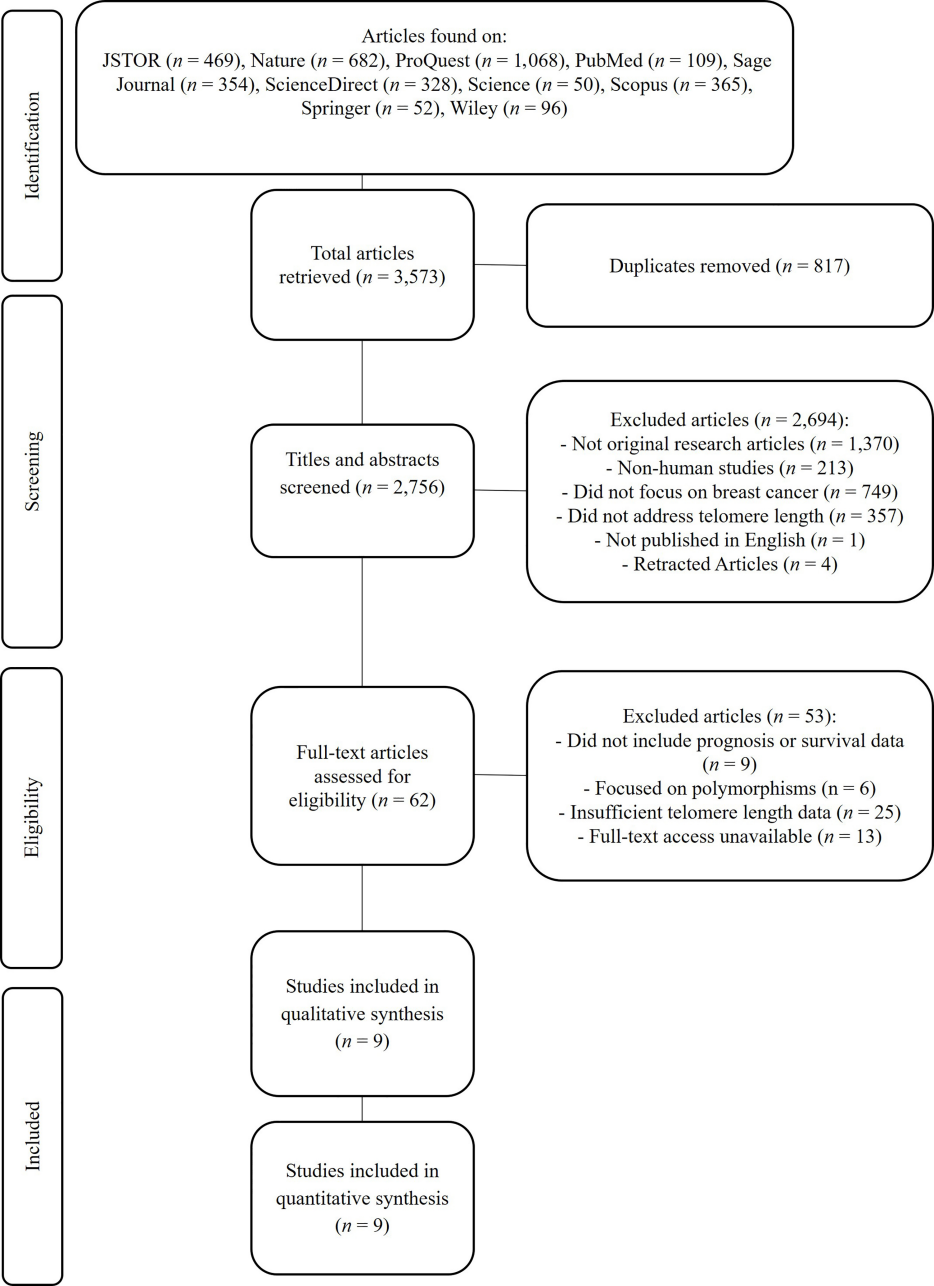
**PRISMA-P flowchart for selecting articles included in systematic reviews and meta-analysis**. PRISMA-P: Preferred Reporting Items for Systematic Reviews and Meta-Analysis Protocols

A systematic search identified 3,573 articles. After removing 817 duplicates, 2,756 titles and abstracts were screened. From these, 62 full-text articles were assessed for eligibility. Nine studies met the inclusion criteria and were included in the final meta-analysis (Figure 1). These studies encompassed 3,145 breast cancer patients [17–25].

### Quality assessment

All included studies scored ≥ 7 on the NOS, indicating a low risk of bias (Table 1). Most studies demonstrated adequate case definitions, robust telomere measurement techniques, and appropriate follow-up durations [30].

**Table 1:** Quality assessment of included studies using the Newcastle-Ottawa Scale

**Study**	**Selection (0–4)**	**Comparability (0–2)**	**Outcome (0–3)**	**Total Score (0–9)**	**Quality rating**
Duggan et al. [17] (2014)	4	2	3	9	High
Fordyce et al. [18] (2006)	4	2	2	8	High
Gay-Bellile et al. [19] (2016)	4	2	3	9	High
Heaphy et al. [20] (2007)	4	2	3	9	High
Lu et al. [21] (2011)	4	2	3	9	High
Shen et al. [23] (2012)	4	2	3	9	High
Simpson et al. [22] (2015)	4	2	3	9	High
Svenson et al. [24] (2008)	4	2	2	8	High
Vodenkova et al. [25] (2020)	4	2	2	8	High

The NOS evaluates study quality based on three main criteria: (1) selection (0–4): assesses the representativeness of the study population, the clarity of case definitions, and the appropriateness of control selection; (2) comparability (0–2): evaluates adjustments for confounding factors in the study design and analysis; (3) outcome (0–3): measures the adequacy of follow-up and the consistency of outcome assessment.

### Study characteristics

The nine included studies varied in geographic regions (America, Europe), sample types (blood plasma, tumor tissue), and telomere measurement methods [relative telomere length (RTL), telomere DNA content]. Follow-up durations ranged from 4.6 to 23 years [17–23], though two studies did not report follow-up periods [24, 25]. Detailed study characteristics are presented in Table 2.

**Table 2:** Characteristics of studies included in the systematic review and meta-analysis

**Author**	**Year**	**Region**	**Sample type**	**Telomere measurement metric**	**Measurement method**	**Patients (*n*)**	**Telomere length**		**Follow-up (years)**	**Cut-off value**	**Outcomes (HR, 95% CI, *P*-value)**
							**Short (*n*)**	**Long (*n*)**			
Duggan et al. [17]	2014	America	Blood plasma	Relative telomere length [telomere-to-single copy gene ratio (T/S ratio)]	qPCR	611	306	305	11.2 years	Median (0.81)	OS: 1.33 (0.90–2.00, *P* = 0.14)
											DSS: 1.33 (0.79–2.27, *P* = 0.27)
Fordyce et al. [18]	2006	America	Tumor tissue	Telomere DNA content (TC)	Slot blot assay	77	35	25	23 years	Tertiles (short < 101%, long > 123%)	DFS: 4.39 (1.47–13.08, *P* = 0.008)
Gay-Bellile et al. [19]	2016	Europe	Tumor tissue	Relative telomere length (T/S ratio)	qPCR	55	22	23	17 years	Median (1.03)	OS: 2.9 (1.00–8.47, *P* = 0.050)
											DFS: 3.31 (1.38–7.04, *P* = 0. 0076)
Heaphy et al. [20]	2007	America	Tumor tissue	TC	Slot blot assay	530	444	86	9.16 years	Threshold (short ≤ 200%, long > 200%)	OS: 2.25 (1.09–4.64, *P* = 0.029)
											DFS: 3.14 (1.27–7.76, *P* = 0.013)
Lu et al. [21]	2011	Europe	Tumor tissue	Relative telomere length (T/S ratio)	qPCR	348	170	166	9 years	Median	OS: 1.27 (0.76–2.13)
											DFS: 1.19 (0.76–1.82)
Shen et al. [23]	2012	America	Blood plasma	Relative telomere length (T/S ratio)	qPCR	1,026	510	516	9.4 years	Median (0.73)	OS: 0.91 (0.68–1.20)
											DSS: 0.99 (0.68–1.45)
Simpson et al. [22]	2015	Europe	Tumor tissue	Telomere length (in kb)	Single telomere length analysis (STELA) assay	120	8	112	4.6 years	Median (2.26 kb)	OS: 21.4 (7.9–57.6, *P* < 0.0001)
Svenson et al. [24]	2008	Europe	Blood plasma	Relative telomere length (T/S ratio)	qPCR	227	114	113	N/A	Median (0.73)	OS: 0.34 (0.16–0.75, *P* = 0.007)
Vodenkova et al. [25]	2020	Europe	Blood plasma	Relative telomere length (T/S ratio)	qPCR	151	N/A	N/A	N/A	Median	OS: 1.02 (0.34–3.05, *P* = 0.97)
											RFS: 0.72 (0.29–1.77, *P* = 0.47)

OS: overall survival; DSS: disease-specific survival; DFS: disease-free survival; RFS: recurrence-free survival; qPCR: quantitative polymerase chain reaction; HR: hazard ratio; CI: confidence intervals; N/A: not available

### Clinicopathological data

Table 3 summarizes the clinicopathological characteristics of breast cancer patients from the nine included studies [17–25]. Tumor grade [20, 21], lymph node metastasis (LNM) status [18, 21], menopausal status [20, 23], and HER2 status [20, 23] were reported in two studies each. Tumor stage [18, 20, 21], estrogen receptor (ER) status [20, 21, 23], and progesterone receptor (PR) status [20, 21, 23] were more frequently reported, appearing in three studies.

**Table 3:** Clinicopathological characteristics of patients with telomere length

**Author**	**Year**	**Tumor grade**		**Tumor stage**		**Menopausal status**		**LNM**		**HER2**		**ER**		**PR**	
		**3**	**1 and 2**	**III and IV**	**I and II**	**Postmenopausal**	**Premenopausal**	**(+)**	**(–)**	**(+)**	**(–)**	**(+)**	**(–)**	**(+)**	**(–)**
Duggan et al. [17]	2014	-	-	-	-	-	-	-	-	-	-	-	-	-	-
Fordyce et al. [18]	2006	-	-	9 (S)/5 (L)	26 (S)/19 (L)	-	-	27 (S)/12 (L)	8 (S)/12 (L)	-	-	-	-	-	-
Gay-Bellile et al. [19]	2016	-	-	-	-	-	-	-	-	-	-	-	-	-	-
Heaphy et al. [20]	2007	90 (S)/14 (L)	204 (S)/43 (L)	5 (S)/0 (L)	428 (S)/84 (L)	299 (S)/59 (L)	131 (S)/25 (L)	-	-	189 (S)/35 (L)	251 (S)/49 (L)	373 (S)/71 (L)	67 (S)/15 (L)	297 (S)/62 (L)	144 (S)/23 (L)
Lu et al. [21]	2011	144 (S)/23 (L)	93 (S)/100 (L)	18 (S)/16 (L)	149 (S)/146 (L)	-	-	74 (S)/83 (L)	94 (S)/79 (L)	-	-	109 (S)/107 (L)	59 (S)/56 (L)	82 (S)/91 (L)	86 (S)/71 (L)
Shen et al. [23]	2012	-	-	-	-	315 (S)/359 (L)	183 (S)/145 (L)	-	-	325 (S)/344 (L)	185 (S)/172 (L)	254 (S)/253 (L)	72 (S)/86 (L)	254 (S)/253 (L)	72 (S)/86 (L)
Simpson et al. [22]	2015	-	-	-	-	-	-	-	-	-	-	-	-	-	-
Svenson et al. [24]	2008	-	-	-	-	-	-	-	-	-	-	-	-	-	-
Vodenkova et al. [25]	2020	-	-	-	-	-	-	-	-	-	-	-	-	-	-

S: short telomere length; L: long telomere length; LNM: lymph node metastasis; HER2: human epidermal growth factor receptor 2; ER: estrogen receptor; PR: progesterone receptor

### Association between telomere length and survival outcomes

#### Overall survival (OS)

**Figure 2:**
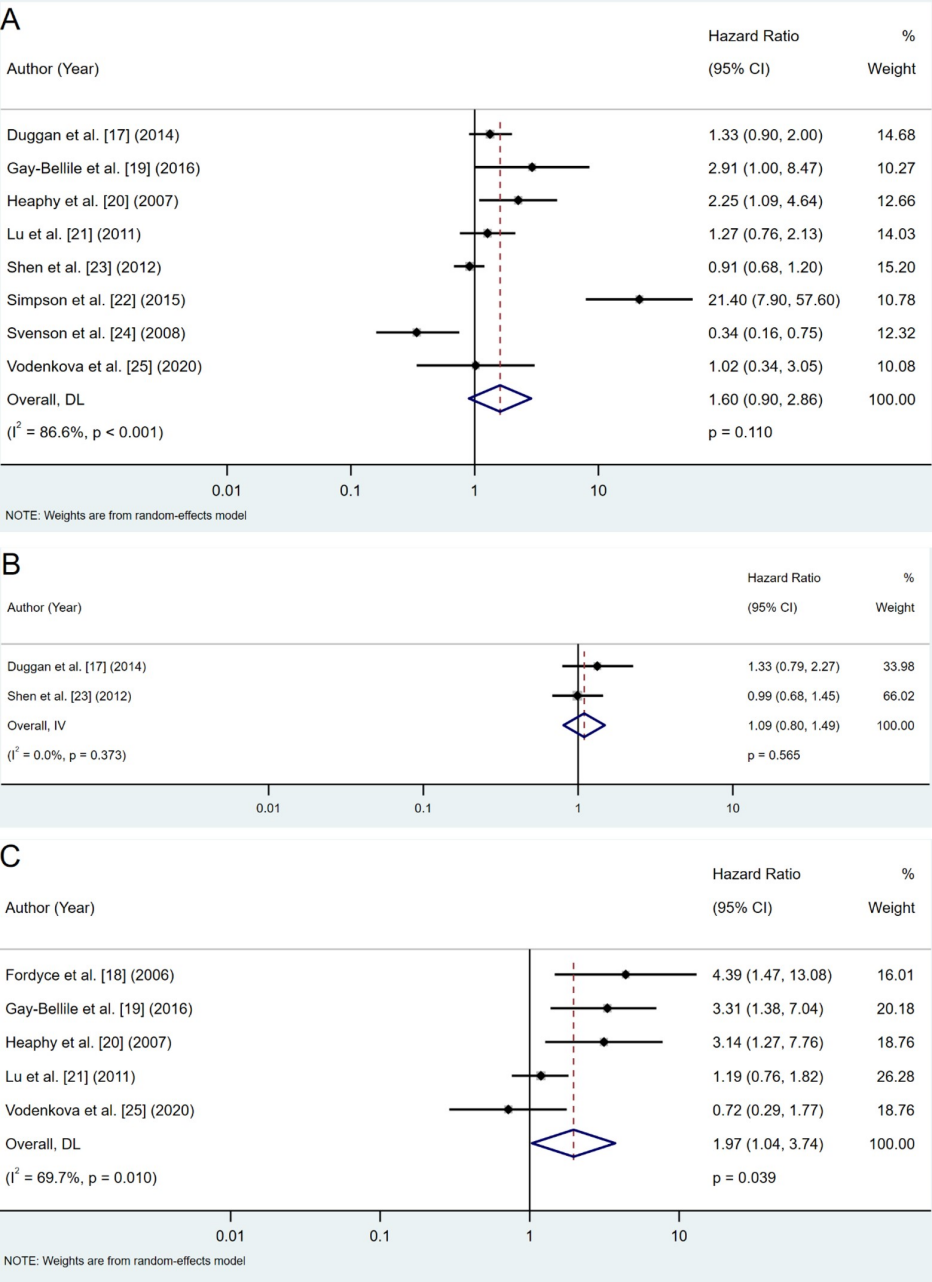
**Forest plots of hazard ratios (HRs) for telomere length and survival outcomes**. **A**. OS analysis; **B**. DSS analysis; **C**. DFS/RFS analysis. DFS: disease-free survival; DSS: disease-specific survival; OS: overall survival; RFS: recurrence-free survival; CI: confidence intervals

Shorter telomere length was associated with a trend toward worse OS across eight studies. However, the result was not statistically significant (pooled HR: 1.60; 95% CI: 0.90–2.86; *P* = 0.110) (Figure 2A).

#### Disease-specific survival (DSS)

Two studies reported DSS outcomes, showing a non-significant trend toward worse DSS in patients with shorter telomeres (pooled HR: 1.09; 95% CI: 0.80–1.49; *P* = 0.565) (Figure 2B).

#### Disease-free survival (DFS) and recurrence-free survival (RFS)

Shorter telomeres were significantly associated with an increased risk of recurrence (pooled HR: 1.97; 95% CI: 1.04–3.74; *P* = 0.039) across five studies (Figure 2C). This indicates a nearly twofold higher risk of recurrence among patients with shorter telomeres.

### Subgroup analyses

**Figure 3:**
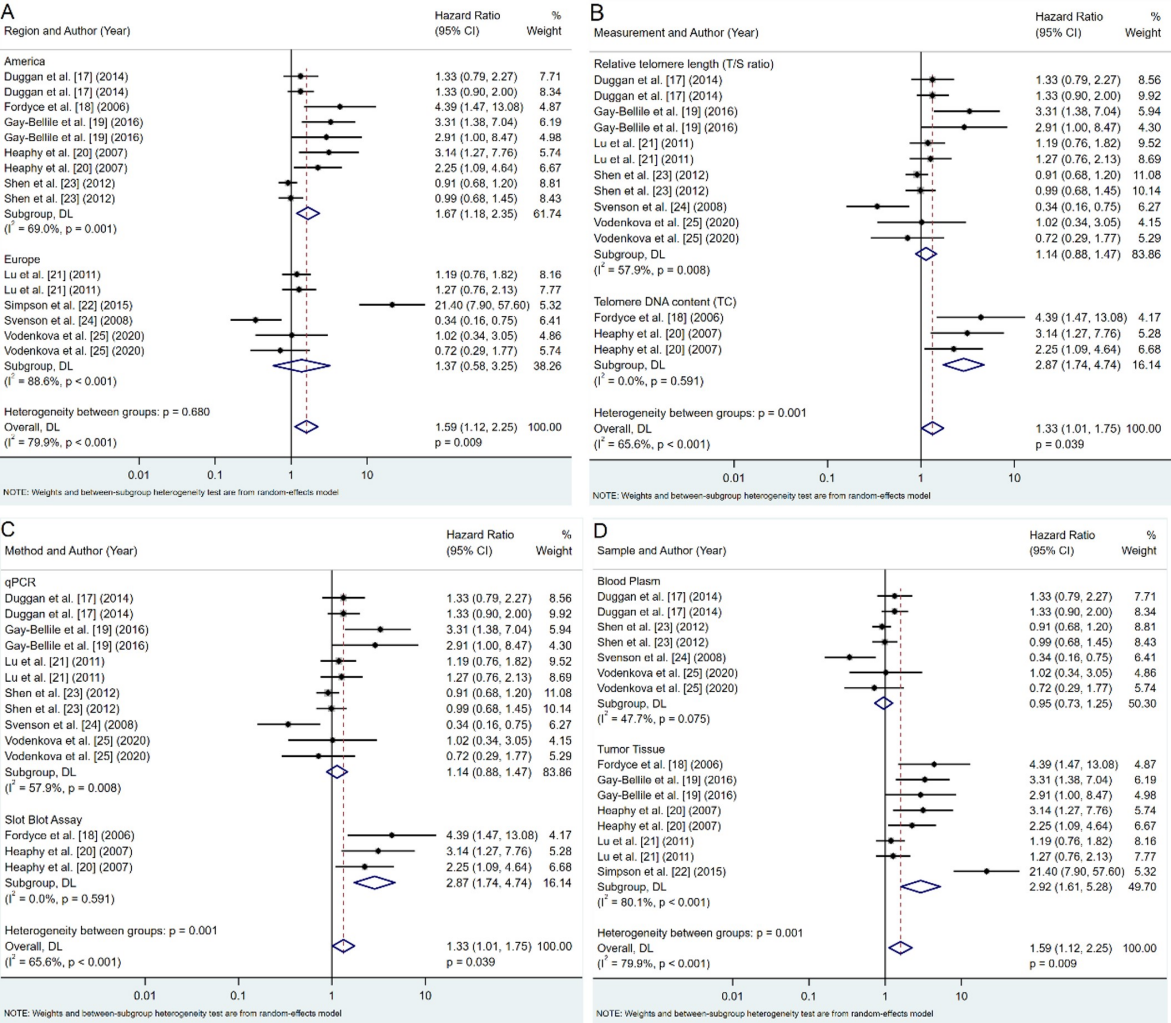
**Subgroup analyses of telomere length and survival outcomes**. **A**. Region; **B**. telomere length measurement metric; **C**. telomere length measurement method; **D**. sample type. CI: confidence intervals

Comprehensive subgroup analyses were conducted to further explore the influence of various factors on the association between telomere length and survival outcomes. These analyses, summarized in Figure 3, examined the impact of geographic location, telomere measurement metrics, methods, and sample type.

#### Geographic locations

Our analysis revealed notable regional differences in the association between telomere length and breast cancer prognosis (Figure 3A). Studies conducted in America showed moderate heterogeneity (*I*^2^ = 69.0%, *P* = 0.001) and a significant association between shorter telomeres and poorer prognosis (pooled HR: 1.67, 95% CI: 1.18–2.35, *P* = 0.003). In contrast, European studies exhibited high heterogeneity (*I*^2^ = 88.6%, *P* < 0.001) and no significant association (pooled HR: 1.37, 95% CI: 0.58–3.25, *P* = 0.468). Despite these apparent differences, the variation between regional subgroups was not statistically significant (*P* = 0.680), suggesting that geographic factors may have a limited impact on the overall conclusions.

#### Telomere measurement metrics

The choice of telomere measurement metric significantly influenced the observed associations (Figure 3B). Telomere DNA content (TC) measurements demonstrated stronger associations with poor prognosis (pooled HR: 2.87; 95% CI: 1.74–4.74; *P* < 0.001) and revealed no heterogeneity (*I*^2^ = 0.0%, *P* = 0.591) compared to RTL [telomere-to-single copy gene ratio (T/S ratio)], which showed no significant association (pooled HR: 1.14; 95% CI: 0.88–1.47; *P* = 0.332) and moderate heterogeneity (*I*^2^ = 57.9%, *P* = 0.008). These findings underscore the importance of selecting robust and reproducible measurement methods, with TC demonstrating a stronger and more consistent association (*P* = 0.001).

#### Telomere measurement methods

A different method of measuring telomeres affects the relationship with survival outcomes (Figure 3C). Studies employing quantitative polymerase chain reaction (qPCR) showed moderate heterogeneity (*I*^2^ = 57.9%, *P* = 0.008) and no significant association with prognosis (pooled HR: 1.14, 95% CI: 0.88–1.47, *P* = 0.332). Meanwhile, the slot blot assay displayed no heterogeneity (*I*^2^ = 0.0%, *P* = 0.591) and a strong association with poor prognosis (pooled HR: 2.87, 95% CI: 1.74–4.74, *P* < 0.001). The differences between these methodological approaches were statistically significant (*P* = 0.001), indicating the potential impact of the measurement method on the observed relationships between telomere length and breast cancer prognosis.

#### Sample type

The type of biological sample used for telomere length measurement also played a crucial role in the observed association (Figure 3D). Telomere length measured in tumor tissue demonstrated a high heterogeneity (*I*^2^ = 80.1%, *P* < 0.001) and a strong correlation with survival outcomes (pooled HR: 2.92; 95% CI: 1.61–5.28; *P* < 0.001). In contrast, blood plasma samples showed low heterogeneity (*I*^2^ = 47.7%, *P* = 0.075) and no significant association with prognosis (pooled HR: 0.95, 95% CI: 0.73–1.25, *P* = 0.734). These results suggest that telomere length measured in tumor tissue is more predictive of survival outcomes than in blood samples, with statistically significant differences between subgroups (*P* = 0.001).

### Association between telomere length and clinicopathological characteristics

**Figure 4:**
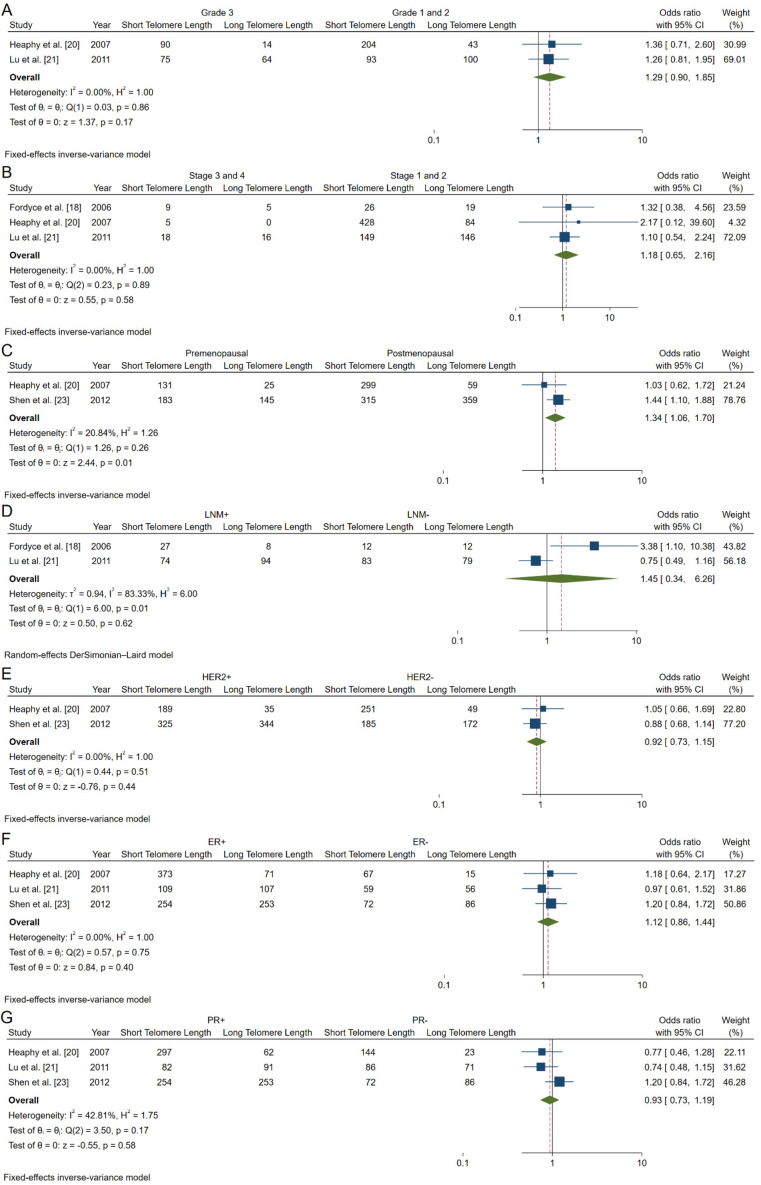
**Forest plots of OR analyzing associations between telomere length and clinicopathological characteristics of breast cancer patients**. **A**. Tumor grade; **B**. tumor stages; **C**. menopausal status; **D**. lymph node involvement status; **E**. HER2 expression; **F**. estrogen receptor (ER); **G**. progesterone receptor (PR). HER2: human epidermal growth factor receptor 2; CI: confidence intervals; LNM: lymph node metastasis; OR: odds ratio

Our meta-analysis investigated the associations between telomere length and various clinicopathological factors in breast cancer patients. The results of these analyses are summarized in Figure 4 and Table 4, which provide a comprehensive overview of the relationships between telomere length and key tumor characteristics.

**Table 4:** Association between telomere length and clinicopathological characteristics of breast cancer patients

**Variable**	**Studies (*n*)**	**Patients (*n*)**	**Telomere length**		**OR (95% CI)**	***P*-value**	**Heterogeneity**		**Model**
			**Short**	**Long**			***I* ^2^ (%)**	***P***	
Tumor grade (3 vs. 1–2)	2	683	165 vs. 297	78 vs. 143	1.29 (0.90–1.85)	0.17	0	0.86	Fixed
Tumor stage (III–IV vs. I–II)	3	905	32 vs. 603	21 vs. 249	1.18 (0.65–2.16)	0.58	0	0.89	Fixed
Menopausal status (premenopausal vs. postmenopausal)	2	1,516	314 vs. 614	170 vs. 418	1.34 (1.06–1.70)	0.01^*^	20.84	0.26	Fixed
Lymph node metastasis (positive vs. negative)	2	389	101 vs. 102	95 vs. 91	1.45 (0.34–6.26)	0.62	83.33	0.01	Random
HER2 (positive vs. negative)	2	1,550	514 vs. 436	379 vs. 221	0.92 (0.73-1.15)	0.44	0	0.51	Fixed
ER (positive vs. negative)	3	1,522	736 vs. 198	431 vs. 157	1.12 (0.86–1.44)	0.40	0	0.75	Fixed
PR (positive vs. negative)	3	1,521	633 vs. 302	406 vs. 180	0.93 (0.73–1.19)	0.58	42.81	0.58	Fixed

^*^ Statistically significant. CI: confidence intervals; ER: estrogen receptor; HER2: human epidermal growth factor receptor 2; OR: odds ratio; PR: progesterone receptor

(1) Tumor grade: Our analysis of tumor grade (Grade 3 vs. Grade 1/2) revealed a non-significant association with shorter telomeres (pooled OR: 1.29; 95% CI: 0.90–1.85; *P* = 0.17) (Figure 4A).
(2) Tumor stage: The association between advanced tumor stage (Stage III/IV vs. Stage I/II) and telomere length was also non-significant (pooled OR: 1.18; 95% CI: 0.65–2.16; *P* = 0.58) (Figure 4B).
(3) Menopausal status: We found a significant association between shorter telomeres and premenopausal status (pooled OR: 1.34; 95% CI: 1.06–1.70; *P* = 0.01) (Figure 4C). This finding suggests that premenopausal breast cancer patients may be more likely to exhibit shorter telomeres, potentially indicating more aggressive tumor biology.
(4) Lymph node involvement: The analysis of lymph node involvement showed a non-significant association with shorter telomeres (pooled OR: 1.45; 95% CI: 0.34–6.26; *P* = 0.62) (Figure 4D).
(5) HER2 status (Figure 4E): Our analysis found no significant association between HER2 status and telomere length (pooled OR: 0.92; 95% CI: 0.73–1.15; *P* = 0.44) (Figure 4E).
(6) ER: The association between ER positivity and telomere length was non-significant (pooled OR: 1.12; 95% CI: 0.86–1.44, *P* = 0.40) (Figure 4F).
(7) PR: PR status showed no significant association with telomere length (pooled OR: 0.93; 95% CI: 0.73–1.19; *P* = 0.58) (Figure 4G).

### Publication bias and sensitivity analysis

**Figure 5:**
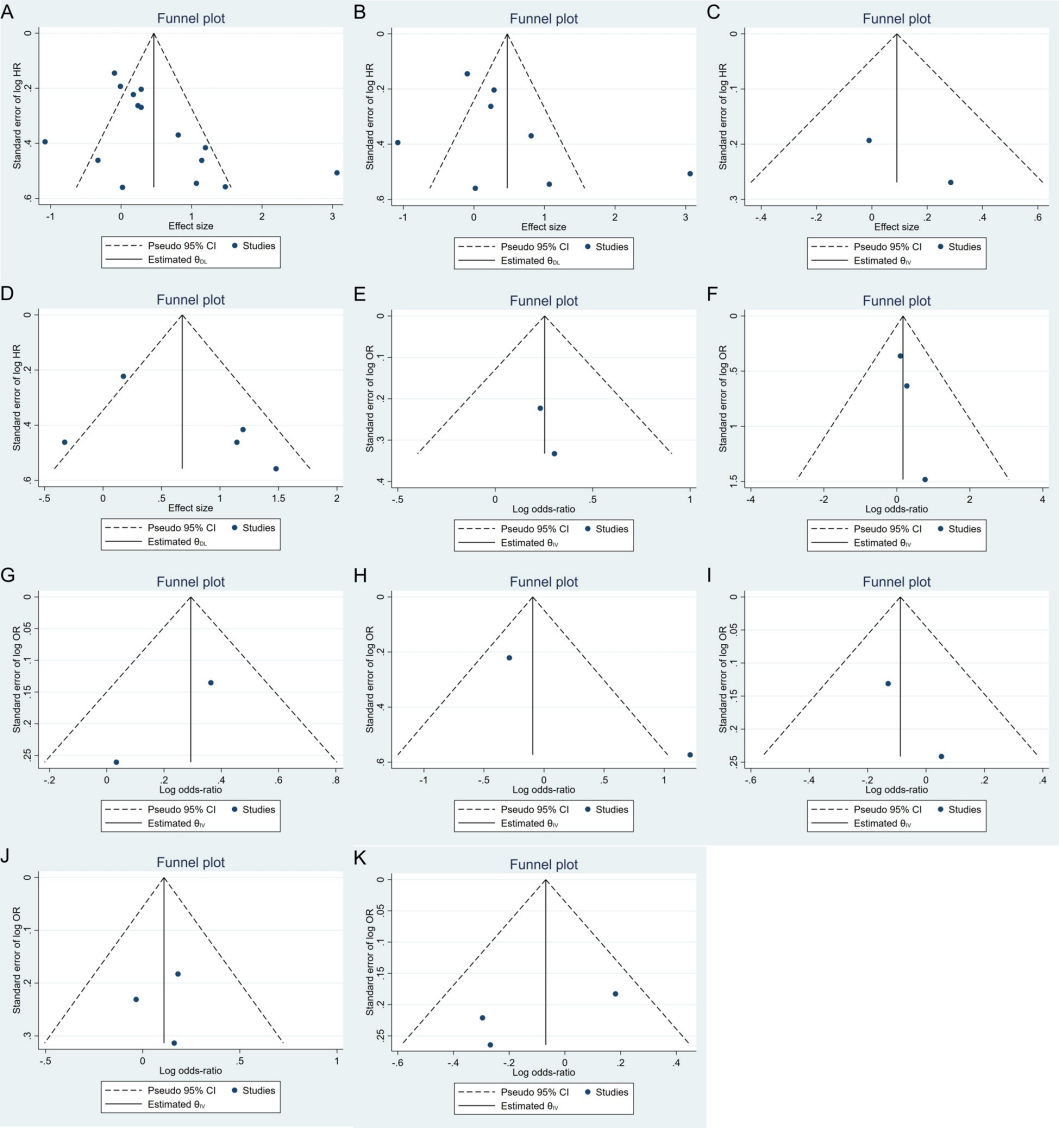
**Funnel plot for assessing publication bias in studies of telomere length and breast cancer prognosis. A**. Survival outcomes; **B**. OS; **C**. DSS; **D**. DFS/RFS; **E**. histopathology; **F**. tumor stage; **G**. menopausal status; **H**. lymph node metastasis; **I**. HER2; **J**. ER; **K**. PR. DFS: disease-free survival; DSS: disease-specific survival; ER: estrogen receptor; HER2: human epidermal growth factor receptor 2; OS: overall survival; PR: progesterone receptor; RFS: recurrence-free survival; HR: hazard ratio; OR: odds ratio; CI: confidence intervals

**Figure 6:**
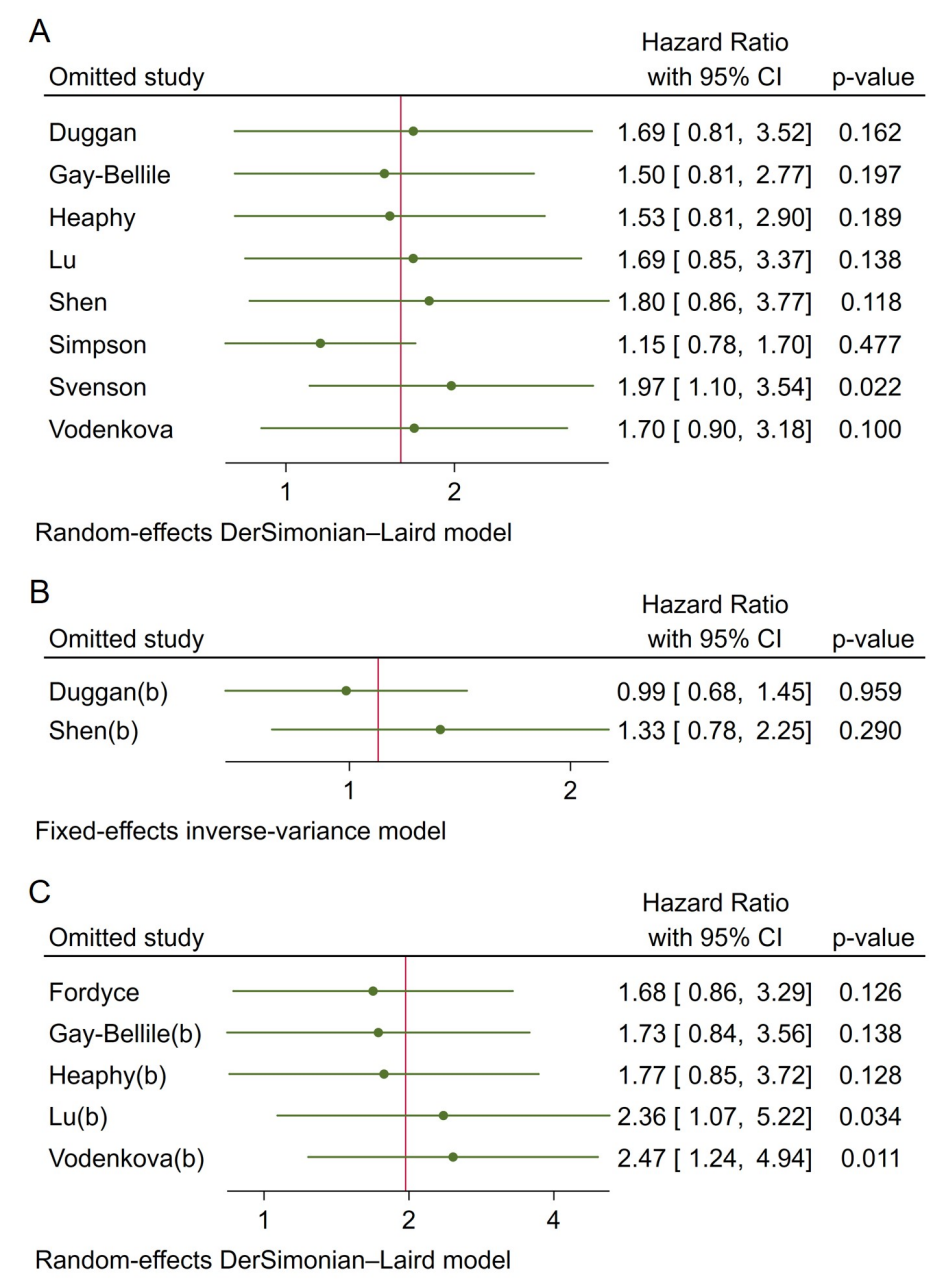
**Sensitivity analysis for pooled hazard ratios**. **A**. OS; **B**. DSS; **C**. DFS/RFS. DFS: disease-free survival; DSS: disease-specific survival; OS: overall survival; RFS: recurrence-free survival; CI: confidence intervals

Publication bias was evaluated using funnel plots and Egger’s and Begg’s tests. The funnel plot (Figure 5) did not indicate significant asymmetry, suggesting no substantial publication bias. Additionally, Egger’s and Begg’s tests (Table 5) showed non-significant results for OS (Egger’s test: 0.1941; Begg’s test: 0.7105) and DFS/RFS (Egger’s test: 0.3944; Begg’s test: 0.2207). However, the limited number of studies warrants cautious interpretation. Sensitivity analysis confirmed that no study disproportionately influenced the results (Figure 6).

**Table 5:** Egger’s and Begg’s test results for publication bias

**Variable**	**Egger’s test (*P* > |t|)**	**Begg’s test (*P* > |z|)**
Survival outcomes	0.0264	0.0600
OS	0.1941	0.7105
DSS	N/A	N/A
DFS/RFS	0.3944	0.2207
Tumor grade	N/A	N/A
Tumor stage	0.7137	0.2963
Menopausal status	N/A	N/A
LNM	N/A	N/A
HER2	0.5094	N/A
ER	0.8970	1.0000
PR	0.3570	1.0000

N/A: not available; DFS: disease-free survival; DSS: disease-specific survival; ER: estrogen receptor; HER2: human epidermal growth factor receptor 2; LNM: lymph node metastasis; OS: overall survival; PR: progesterone receptor; RFS: recurrence-free survival

## Discussion

This meta-analysis provides a comprehensive evaluation of telomere length as a prognostic biomarker for survival outcomes in breast cancer, specifically focusing on OS, DSS, DFS, and RFS. Our findings indicate that shorter telomere length is significantly associated with increased recurrence risk (DFS/RFS) (pooled HR: 1.97; 95% CI: 1.04–3.74; *P* = 0.039), indicating a nearly twofold increase in recurrence risk for patients with shorter telomeres. While trends toward worse OS (pooled HR: 1.60; 95% CI: 0.90–2.86, *P* = 0.110) and DSS (pooled HR: 1.09; 95% CI: 0.80–1.49, *P* = 0.565) were observed for shorter telomeres, these associations did not reach statistical significance. These findings reinforce the emerging role of telomere length as a valuable prognostic marker in breast cancer, with shorter telomeres linked to poorer outcomes and altered telomerase activity, further emphasizing the role of telomere dynamics in disease progression [35, 36].

Telomere shortening affects genomic stability and promotes cancer recurrence by increasing chromosomal instability, activating oncogenes, and inactivating tumor suppressor genes [10, 37]. This instability accelerates cell proliferation and malignant transformation, contributing to higher recurrence rates after treatment [10, 37]. While telomere shortening can trigger cell cycle arrest as a protective mechanism against tumorigenesis, senescent cells can also create a pro-tumorigenic microenvironment. Through the senescence-associated secretory phenotype (SASP), these cells promote inflammation and alter the tissue microenvironment, further influencing recurrence [38]. The role of telomere shortening in genomic instability likely explains the higher recurrence rates observed in patients with shorter telomeres [18–21], although one study reports differing results [25]. These inconsistencies may arise from variations in telomere measurement techniques, sample types, and patient characteristics.

Subgroup analyses revealed important insights regarding the influence of telomere measurement methods. TC showed stronger associations with prognosis (pooled HR: 2.87; 95% CI: 1.74–4.74; *P* < 0.001) than RTL (T/S ratio). The slot blot assay also demonstrated a stronger correlation with poor prognosis (pooled HR: 2.87, 95% CI: 1.74–4.74, *P* < 0.001) compared to qPCR. Southern blotting remains the most accurate and reliable method for precise telomere length measurement. While qPCR is faster, it is less precise and may not capture specific telomere dynamics, such as the shortest telomeres or sub-telomeric regions, highlighting the need for caution when using qPCR, especially in longitudinal studies [39, 40]. These findings underscore the importance of telomere measurement methodology and suggest that absolute telomere length measurements could be more informative for prognostic purposes [41].

Moreover, telomere length measured in tumor tissue exhibited a stronger association with survival outcomes (pooled HR: 2.92; 95% CI: 1.61–5.28; *P* < 0.001) than measurements from blood plasma samples. Telomere shortening is more pronounced in tumor tissues than in adjacent non-cancerous tissues, reflecting cancer-specific dynamics more accurately than plasma telomeric cfDNA, which may be influenced by systemic factors [42, 43]. Tumor tissue telomeres are typically shorter due to the high proliferative activity and genomic instability of cancer cells, emphasizing the importance of tissue-specific telomere dynamics [43, 44]. While liquid biopsy methods, such as plasma cfDNA are non-invasive, they may miss localized telomere changes seen in tumor tissues, which offer a more accurate reflection of the tumor microenvironment [43]. This highlights the need to carefully consider sample types in future studies and clinical applications.

The geographic variability observed in our analysis, with American studies showing a significant association between shorter telomeres and poorer prognosis (pooled HR: 1.67, 95% CI: 1.18–2.35, *P* = 0.003), while European studies did not, warrants further investigation. This discrepancy may stem from genetic variation, geographic factors, and socioeconomic conditions that influence cancer risk and prognosis [45, 46]. These findings emphasize the importance of considering regional variations in telomere biology research.

Additionally, the association between shorter telomeres and premenopausal status (pooled OR: 1.34, 95% CI: 1.06–1.70, *P* = 0.01), suggests that telomere length may be particularly relevant for younger breast cancer patients. A case-control study in a Chinese Han population found a significant link between shorter telomeres and increased breast cancer risk, supporting the role of telomere length as a potential biomarker for breast cancer susceptibility in premenopausal women [47]. Furthermore, research on prediagnostic leukocyte telomere length has demonstrated an association with breast cancer risk, reinforcing the importance of telomere length as an indicator for premenopausal breast cancer [48]. Another study highlighted that shorter RTL was associated with increased breast cancer risk, although it primarily focused on postmenopausal women. These findings collectively broaden the context, reinforcing the relevance of telomere length as a potential biomarker for breast cancer risk across different populations [49]. Future studies should focus on validating telomere length as a prognostic marker, particularly in this high-risk subgroup.

Our analysis confirms the significant association between shorter telomere length and poorer survival outcomes in breast cancer patients. Telomere dysfunction, which leads to genomic instability by impairing DNA repair mechanisms, increasing oxidative stress, and promoting chromosomal fusions, has been widely recognized as a critical factor in cancer progression [7–9, 50]. This instability can contribute to tumor growth and recurrence, aligning with our findings that shorter telomeres correlate with worse DFS and RFS.

While the mechanisms underlying telomere dysfunction are well understood, the clinical utility of telomere length as a biomarker remains limited due to variability in measurement techniques. Studies have shown that the lack of standardized telomere measurement protocols is a key barrier to its widespread clinical application [26]. This underscores the need for future research to address these challenges and establish standardized protocols to enhance the reliability and applicability of telomere length as a prognostic biomarker for breast cancer.

The relationship between telomere dysfunction and *BRCA2* mutations also warrants further exploration. Since *BRCA2* mutations impair DNA repair mechanisms, telomere shortening may play a particularly critical role in increasing the risk of cancer progression in patients with this genetic predisposition [27]. This could potentially open up opportunities for using telomere length as an additional marker for identifying high-risk breast cancer patients, particularly those with hereditary breast cancer linked to *BRCA* mutations.

Despite its strengths, this meta-analysis has limitations. The small number of included studies reduces the generalizability of the findings and limits the statistical power of subgroup analyses, particularly for DSS and certain clinicopathological characteristics. The reliance on retrospective data may introduce selection bias, and the precision of HRs estimated from Kaplan-Meier curves is inherently limited compared to directly reported values [29]. Furthermore, while no significant publication bias was detected, the small sample size warrants cautious interpretation of these results.

In conclusion, our meta-analysis provides evidence for the potential of telomere length as a prognostic biomarker in breast cancer, particularly for predicting recurrence risk. The prognostic value of telomere length appears to be influenced by measurement method and sample type, highlighting the need for standardization. While challenges remain in translating these findings into clinical practice, the integration of telomere biology into personalized medicine approaches holds promise for improving breast cancer management. Future research should focus on addressing methodological inconsistencies, exploring telomere-based therapies, and validating the clinical utility of telomere length measurements in large-scale, prospective studies.

## Abbreviations

CI: confidence intervals
DFS: disease-free survival
DSS: disease-specific survival
ER: estrogen receptor
HER2: human epidermal growth factor receptor 2
HRs: hazard ratios
LNM: lymph node metastasis
NOS: Newcastle-Ottawa Scale
OR: odds ratio
OS: overall survival
PR: progesterone receptor
PRISMA-P: Preferred Reporting Items for Systematic Reviews and Meta-Analysis Protocols
PROSPERO: International Prospective Register of Systematic Reviews
qPCR: quantitative polymerase chain reaction
RFS: recurrence-free survival
RTL: relative telomere length
T/S ratio: telomere-to-single copy gene ratio
TC: telomere DNA content
TNBC: triple-negative breast cancer

## Supplementary materials

The supplementary material for this article is available at: https://www.explorationpub.com/uploads/Article/file/1002289_sup_1.pdf.

## Declarations

### Availability of data and materials

All data generated or analyzed for this study are included in the manuscript and the supplementary files.

### Copyright

© The Author(s) 2025.

## References

[1] Sung H, Ferlay J, Siegel RL, Laversanne M, Soerjomataram I, Jemal A (2021). Global Cancer Statistics 2020: GLOBOCAN Estimates of Incidence and Mortality Worldwide for 36 Cancers in 185 Countries. CA Cancer J Clin.

[2] Lüönd F, Tiede S, Christofori G (2021). Breast cancer as an example of tumour heterogeneity and tumour cell plasticity during malignant progression. Br J Cancer.

[3] Łukasiewicz S, Czeczelewski M, Forma A, Baj J, Sitarz R, Stanisławek A (2021). Breast Cancer-Epidemiology, Risk Factors, Classification, Prognostic Markers, and Current Treatment Strategies-An Updated Review. Cancers (Basel).

[4] Smolarz B, Nowak AZ, Romanowicz H (2022). Breast Cancer-Epidemiology, Classification, Pathogenesis and Treatment (Review of Literature). Cancers (Basel).

[5] Zhang C, Chen X, Li L, Zhou Y, Wang C, Hou S (2015). The Association between Telomere Length and Cancer Prognosis: Evidence from a Meta-Analysis. PLoS One.

[6] Srinivas N, Rachakonda S, Kumar R (2020). Telomeres and Telomere Length: A General Overview. Cancers (Basel).

[7] Barnes RP, Fouquerel E, Opresko PL (2019). The impact of oxidative DNA damage and stress on telomere homeostasis. Mech Ageing Dev.

[8] Moustakli E, Zikopoulos A, Sakaloglou P, Bouba I, Sofikitis N, Georgiou I (2023). Functional association between telomeres, oxidation and mitochondria. Front Reprod Health.

[9] Rossiello F, Jurk D, Passos JF, d’Adda di Fagagna F (2022). Telomere dysfunction in ageing and age-related diseases. Nat Cell Biol.

[10] Okamoto K, Seimiya H (2019). Revisiting Telomere Shortening in Cancer. Cells.

[11] Dos Santos GA, Viana NI, Pimenta R, de Camargo JA, Guimaraes VR, Romão P (2024). Upregulation of shelterin and CST genes and longer telomeres are associated with unfavorable prognostic characteristics in prostate cancer. Cancer Genet.

[12] Ceja-Rangel HA, Sánchez-Suárez P, Castellanos-Juárez E, Peñaroja-Flores R, Arenas-Aranda DJ, Gariglio P (2016). Shorter telomeres and high telomerase activity correlate with a highly aggressive phenotype in breast cancer cell lines. Tumour Biol.

[13] Kammori M, Sugishita Y, Okamoto T, Kobayashi M, Yamazaki K, Yamada E (2015). Telomere shortening in breast cancer correlates with the pathological features of tumor progression. Oncol Rep.

[14] Murillo-Ortiz BO, García-Corrales K, Martínez-Garza S, Romero-Vázquez MJ, Agustín-Godínez E, Escareño-Gómez A (2024). Association of hTERT expression, Her2Neu, estrogen receptors, progesterone receptors, with telomere length before and at the end of treatment in breast cancer patients. Front Med (Lausanne).

[15] Lin F, Huang J, Zhu W, Jiang T, Guo J, Xia W (2023). Prognostic value and immune landscapes of TERT promoter methylation in triple negative breast cancer. Front Immunol.

[16] Yang L, Wang B, Jiao X, Zhou C, Chen S, Gao X (2021). TAZ maintains telomere length in TNBC cells by mediating Rad51C expression. Breast Cancer Res.

[17] Duggan C, Risques R, Alfano C, Prunkard D, Imayama I, Holte S (2014). Change in peripheral blood leukocyte telomere length and mortality in breast cancer survivors. J Natl Cancer Inst.

[18] Fordyce CA, Heaphy CM, Bisoffi M, Wyaco JL, Joste NE, Mangalik A (2006). Telomere content correlates with stage and prognosis in breast cancer. Breast Cancer Res Treat.

[19] Gay-Bellile M, Romero P, Cayre A, Véronèse L, Privat M, Singh S (2016). ERCC1 and telomere status in breast tumours treated with neoadjuvant chemotherapy and their association with patient prognosis. J Pathol Clin Res.

[20] Heaphy CM, Baumgartner KB, Bisoffi M, Baumgartner RN, Griffith JK (2007). Telomere DNA content predicts breast cancer-free survival interval. Clin Cancer Res.

[21] Lu L, Zhang C, Zhu G, Irwin M, Risch H, Menato G (2011). Telomerase expression and telomere length in breast cancer and their associations with adjuvant treatment and disease outcome. Breast Cancer Res.

[22] Simpson K, Jones RE, Grimstead JW, Hills R, Pepper C, Baird DM (2015). Telomere fusion threshold identifies a poor prognostic subset of breast cancer patients. Mol Oncol.

[23] Shen J, Gammon MD, Terry MB, Bradshaw PT, Wang Q, Teitelbaum SL (2012). Genetic polymorphisms in telomere pathway genes, telomere length, and breast cancer survival. Breast Cancer Res Treat.

[24] Svenson U, Nordfjäll K, Stegmayr B, Manjer J, Nilsson P, Tavelin B (2008). Breast cancer survival is associated with telomere length in peripheral blood cells. Cancer Res.

[25] Vodenkova S, Kroupa M, Polivkova Z, Musak L, Ambrus M, Schneiderova M (2020). Chromosomal damage and telomere length in peripheral blood lymphocytes of cancer patients. Oncol Rep.

[26] Ferrer A, Stephens ZD, Kocher JA (2023). Experimental and Computational Approaches to Measure Telomere Length: Recent Advances and Future Directions. Curr Hematol Malig Rep.

[27] Thorvaldsdottir B, Aradottir M, Stefansson OA, Bodvarsdottir SK, Eyfjörd JE (2017). Telomere Length Is Predictive of Breast Cancer Risk in *BRCA2* Mutation Carriers. Cancer Epidemiol Biomarkers Prev.

[28] Page MJ, Moher D, Bossuyt PM, Boutron I, Hoffmann TC, Mulrow CD (2021). PRISMA 2020 explanation and elaboration: updated guidance and exemplars for reporting systematic reviews. BMJ.

[29] Tierney JF, Stewart LA, Ghersi D, Burdett S, Sydes MR (2007). Practical methods for incorporating summary time-to-event data into meta-analysis. Trials.

[30] Stang A (2010). Critical evaluation of the Newcastle-Ottawa scale for the assessment of the quality of nonrandomized studies in meta-analyses. Eur J Epidemiol.

[31] Halme ALE, McAlpine K, Martini A (2023). Fixed-effect Versus Random-effects Models for Meta-analyses: Random-effects Models. Eur Urol Focus.

[32] Sterne JA, Egger M, Smith GD (2001). Systematic reviews in health care: Investigating and dealing with publication and other biases in meta-analysis. BMJ.

[33] Egger M, Davey Smith G, Schneider M, Minder C (1997). Bias in meta-analysis detected by a simple, graphical test. BMJ.

[34] Begg CB, Mazumdar M (1994). Operating characteristics of a rank correlation test for publication bias. Biometrics.

[35] Ennour-Idrissi K, Maunsell E, Diorio C (2017). Telomere Length and Breast Cancer Prognosis: A Systematic Review. Cancer Epidemiol Biomarkers Prev.

[36] Benites-Zapata VA, Ulloque-Badaracco JR, Alarcón-Braga EA, Fernández-Alonso AM, López-Baena MT, Pérez-López FR (2024). Telomerase activity and telomere length in women with breast cancer or without malignancy: A systematic review and meta-analysis. Maturitas.

[37] Maciejowski J, de Lange T (2017). Telomeres in cancer: tumour suppression and genome instability. Nat Rev Mol Cell Biol.

[38] Yang J, Liu M, Hong D, Zeng M, Zhang X (2021). The Paradoxical Role of Cellular Senescence in Cancer. Front Cell Dev Biol.

[39] Lindrose AR, McLester-Davis LWY, Tristano RI, Kataria L, Gadalla SM, Eisenberg DTA (2021). Method comparison studies of telomere length measurement using qPCR approaches: A critical appraisal of the literature. PLoS One.

[40] Aviv A, Hunt SC, Lin J, Cao X, Kimura M, Blackburn E (2011). Impartial comparative analysis of measurement of leukocyte telomere length/DNA content by Southern blots and qPCR. Nucleic Acids Res.

[41] Lai TP, Wright WE, Shay JW (2018). Comparison of telomere length measurement methods. Philos Trans R Soc Lond B Biol Sci.

[42] Wu X, Tanaka H (2015). Aberrant reduction of telomere repetitive sequences in plasma cell-free DNA for early breast cancer detection. Oncotarget.

[43] Holesova Z, Krasnicanova L, Saade R, Pös O, Budis J, Gazdarica J (2023). Telomere Length Changes in Cancer: Insights on Carcinogenesis and Potential for Non-Invasive Diagnostic Strategies. Genes (Basel).

[44] Looi LM, Cheah PL, Ng MH, Yip CH, Mun KS, Rahman NA (2010). Comparison of telomere length and telomerase activation between breast fibroadenoma and infiltrating ductal carcinoma in Malaysian women. Asian Pac J Cancer Prev.

[45] Alexeeff SE, Schaefer CA, Kvale MN, Shan J, Blackburn EH, Risch N (2019). Telomere length and socioeconomic status at neighborhood and individual levels among 80,000 adults in the Genetic Epidemiology Research on Adult Health and Aging cohort. Environ Epidemiol.

[46] Hunt SC, Hansen MEB, Verhulst S, McQuillan MA, Beggs W, Lai TP (2020). Genetics and geography of leukocyte telomere length in sub-Saharan Africans. Hum Mol Genet.

[47] Wang Z, Zhang Z, Guo Y, Shui H, Liu G, Jin T (2018). Shorter Telomere Length Is Associated with Increased Breast Cancer Risk in a Chinese Han Population: A Case-Control Analysis. J Breast Cancer.

[48] Samavat H, Xun X, Jin A, Wang R, Koh WP, Yuan JM (2019). Association between prediagnostic leukocyte telomere length and breast cancer risk: the Singapore Chinese Health Study. Breast Cancer Res.

[49] De Vivo I, Prescott J, Wong JYY, Kraft P, Hankinson SE, Hunter DJ (2009). A Prospective Study of Relative Telomere Length and Postmenopausal Breast Cancer Risk. Cancer Epidemiol Biomarkers Prev.

[50] Karimi Forood AM (2024). Mechanisms of telomere dysfunction in cancer from genomic instability to therapy: A review. Int J Sci Res Arc.

