# Supplementary material for: Shorter telomere length as a prognostic marker for survival and recurrence in breast cancer: a systematic review and meta-analysis
Source: Explor Target Antitumor Ther. 2025 Feb 13;6:1002289. doi: 10.37349/etat.2025.1002289 (PMC11886381; doi:10.37349/etat.2025.1002289)
Supplement: Supplementary file 1 [file 1002289_sup_1.pdf]

Table S1. Keyword Search Strategy for Each Database

Database	Search Terms	Articles Retrieved
**JSTOR**	(((Telomere Length) AND ((Breast Cancer) OR (Carcinoma Mammae))) AND ((Prognos*) OR (Surviv*) OR (Recurr*)))	**469**
**Nature**	((Telomere Length)) AND ((Prognos*) OR (Surviv*) OR (Recurr*)) AND ((Breast Neoplasm) OR (Breast Tumor) OR (Breast Cancer) OR (Breast Malignant Neoplasms) OR (Mammary Carcinoma))	**682**
**ProQuest**	((Telomere Length)) AND ((Prognos*) OR (Surviv*) OR (Recurr*)) AND ((Breast Neoplasm) OR (Breast Tumor) OR (Breast Cancer) OR (Mammary Cancer) OR (Breast Malignant Neoplasms) OR (Mammary Carcinoma) OR (Breast Carcinoma))	**1068**
**PubMed**	(((((((Breast Neoplasm) OR (Breast Tumor)) OR (Mammary Cancer)) OR (Breast Malignant Neoplasm)) OR (Mammary Carcinoma)) OR (Breast Cancer)) AND (Telomere Length)) AND (((Prognosis) OR (Survival)) OR (Recurrence))	**109**
**Sage Journals**	((Telomere Length)) AND ((Prognos*) OR (Surviv*) OR (Recurr*)) AND ((Breast Neoplasm) OR (Breast Tumor) OR (Breast Cancer) OR (Mammary Cancer) OR (Breast Malignant Neoplasms) OR (Mammary Carcinoma) OR (Breast Carcinoma))	**354**
**ScienceDirect**	(((Telomere Length)) AND ((Prognosis) OR (Survival) OR (Recurrence))) Title, abstract, (((Breast Neoplasm) OR (Breast Tumor) OR (Breast Cancer) OR (Breast Malignant Neoplasms) OR (Mammary Carcinoma)))	**328**

**Science**	((Telomere Length)) AND ((Prognos*) OR (Surviv*) OR (Recurr*)) AND ((Breast Neoplasm) OR (Breast Tumor) OR (Breast Cancer) OR (Breast Malignant Neoplasms) OR (Mammary Carcinoma))	**50**
**Scopus**	((TITLE-ABS-KEY (breast AND neoplasm) OR TITLE-ABS-KEY (breast AND tumor) OR TITLE-ABS-KEY (breast AND cancer) OR TITLE-ABS-KEY (breast AND malignant AND neoplasms) OR TITLE-ABS-KEY (mammary AND carcinoma))) AND (TITLE-ABS-KEY (telomere AND length)) AND ((ALL (prognos*) OR ALL (surviv*) OR ALL (recurr*)))	**365**
**Springer**	((Telomere Length)) AND ((Prognos*) OR (Surviv*) OR (Recurr*)) AND ((Breast Neoplasm) OR (Breast Tumor) OR (Breast Cancer) OR (Mammary Cancer) OR (Breast Malignant Neoplasms) OR (Mammary Carcinoma) OR (Breast Carcinoma))	**52**
**Wiley**	((Telomere Length))" anywhere and "((Prognos*) OR (Surviv*) OR (Recurr*))" anywhere and "((Breast Neoplasm) OR (Breast Tumor) OR (Breast Cancer) OR (Mammary Cancer) OR (Breast Malignant Neoplasms) OR (Mammary Carcinoma) OR (Breast Carcinoma))" in Title	**96**
